# Kansas City Veterinary College

**Published:** 1897-06

**Authors:** 


### COMMENCEMENT EXERCISES.

### KANSAS CITY VETERINARY COLLEGE.

THIS college closed its session on Wednesday, March 31st. The exercises were held in the College Hall, and proved a very pleasant innovation, as they were confined to those who were specially interested in the school and this year's graduates.

The following members of the class of '97 received the degree of the college: George R. Conrad, Robert B. Leeper, James M. Moore, Frank A. Pouppirt, John B. Wright; and L. E. Day, V.S., and H. Ovens, V.S., who had attended the post-graduate course in milk- and meat-inspection were granted evidence of having pursued the same. The exercises were completed at the Savoy Hotel at 8.30 P.M., where, after presentation of the diplomas by Dr. C. J. Sihler, the class response was made by Dr. R. B. Leeper; the toast of the Kansas City Veterinary College by Dr. S. Stewart; "My Friend, the Quack," Dr. S. L. Brooking; "One Year in Practice," Dr. S. Sheldon, Lathrop, Mo.; "The Veterinary and Medical Professions." Dr. L. Rosenwald; "The Veterinarian under Cross-examination," Mr. Frank Sheridan; "The Veterinarian's Place in Society," Dr. R. H. Harrison. The toastmaster of the occasion was Dr. I. J. Wolf, who filled well the place assigned him.

All enjoyed the excellent repast provided for those who were privileged to be seated at the table.

